# Septic arthritis complicating *Streptobacillus moniliformis* rat bite fever: a case report and review of its pathophysiology and diagnosis

**DOI:** 10.3389/fmed.2024.1345354

**Published:** 2024-08-29

**Authors:** Emmanuelle Giraudon, Eva Larranaga Lapique, Silvio Wallemacq, Marie Dalborgo, Nicolas Yin, Maya Hites, Delphine Martiny

**Affiliations:** ^1^Department of Microbiology, Laboratoire Hospitalier Universitaire de Bruxelles-Brussel Universitair Laboratorium (LHUB-ULB), Université Libre de Bruxelles (ULB), Brussels, Belgium; ^2^Clinique des Maladies Infectieuses, Hôpital Universitaire de Bruxelles (HUB), Université Libre de Bruxelles, Brussels, Belgium; ^3^Faculty of Medicine and Pharmacy, University of Mons (UMONS), Mons, Belgium

**Keywords:** *Streptobacillus moniliformis*, rat bite fever, septic arthritis, asymmetric polyarthritis, rodent zoonosis, discitis, microbiological diagnosis, seronegative polyarthritis

## Abstract

Rat bite fever is characterized by a clinical triad of symptoms, fever, rash and arthritis. It is transmitted by rodents and mainly due to infection by *Streptobacillus moniliformis*, a fastidious bacterium carried by *Rattus norvegicus*. This case report presents the case of a patient who developed septic arthritis and fever after a wild rat bite, with subsequent isolation of *S. moniliformis* from the joint fluid. Upon reviewing 45 other published case reports of *S. moniliformis* osteoarticular infections following contact with either a rat or its secretions, it was firstly observed that the rat bite fever clinical triad was incomplete in over half of the cases, mainly because rash was infrequently observed among adult patients. Secondly, the clinical presentation of rat bite fever is quite non-specific and rodent exposure is not mentioned by patients in a third of cases upon admission. Altogether, diagnosing rat bite fever is a significant clinical challenge suggesting that it might be significantly underdiagnosed. In addition to these clinical aspects, no evidence was found supporting immunological mechanisms, as suggested in some literature. Instead, when excluding five improperly performed cultures, *S. moniliformis* was cultured in 25 reported cases and identified twice by direct PCR sequencing amounting to a detection rate of 90% (*n* = ^27^/_30_) on joint fluids. Cultures should be performed in medium containing yeast extract, complete peptic digest of animal tissue and at least 5% blood. Knowing that *S. moniliformis* is very sensitive to many antibiotics thereby making the culture negative, direct 16S rRNA gene sequencing on joint fluid is an alternative method in the case of clinical and cytological evidence of osteoarticular infections with sterile culture of joint fluid.

## Introduction

Rodents are reservoirs for more than 60 zoonotic diseases (1), some of which can be life-threatening. The recognition of diseases resulting from rat bites dates back more than 2,000 years (2, 3). However, formal reports of illnesses linked to rat bites did not emerge until 1839 in the USA and were described as “violent symptoms from the bite of a rat” (4). In 1914, *Streptothrix muris ratti* was isolated from a man who was bitten by a rat (5), and later renamed *Streptobacillus moniliformis* in 1925 (6).

In 1926, Haverhill, Massachusetts, experienced an outbreak of a disease termed “Haverhill fever” (HF) which was associated with contaminated raw milk consumption (7). After an incubation period of 2 to 3 days, patients typically presented a triad of symptoms: sudden onset of recurrent fever (96.5% of the cases), rash (93%), and delayed, extremely painful, persistent and disabling polyarthritis (96.5%). Patients also experienced a sore throat (67%), vomiting (62%), and coughing (24%). The causative organism, found in blood samples and infected joint fluids, was initially taxonomically designated *Haverhillia multiformis* (8) but was later confirmed to be identical to *S. moniliformis* (3, 9).

In 1983, another outbreak of HF occurred in a school in the United Kingdom, affecting 304 children (10–12) which was linked to the water from a spring pond near which rats were observed (12). All studied patients exhibited fever, 97% experienced arthritis affecting several joints in 87% of cases and 95% developed a rash (12).

Alongside these HF outbreaks, numerous cases of rat bite fever (RBF) have been documented and reviewed until 2023 (3, 13–20). In 2001 Graves et al. (15) conducted a retrospective study of 41 RBF cases that had occurred in California over the past three decades finding 88% of patients presenting fever but only 73% exhibiting arthritis and/or arthralgia and 65% developing rash. In a study by Elliot (17), it was reported that out of 65 published cases, 92% of patients had fever. However only 61% presented with a rash and 66% experienced polyarthralgias. Furthermore, sore throat and vomiting were reported in fewer than half of the cases. In both studies, the primary route of transmission was a rat bite, followed by direct exposure to rat secretions. These observations align with those of Ojukwu and Christy (16) who reviewed 12 pediatric cases and Hadvani et al. (18) who presented 12 cases from a children's hospital in Houston, USA: among both groups only one third (4/12) of patients presented the typical triad of symptoms with fever being the most frequent manifestation and rash being the least frequent one. Focusing on patients displaying *S. moniliformis* septic arthritis, Dendle et al. (21) found that 87.5% of 16 patients had fever but only 25% of them exhibited a rash, a finding corroborated by Adams and Mahapatra (22).

Given these partially consistent clinical observations of *S. moniliformis* infections spanning the past century, this case report presents a new case of *S. moniliformis* septic arthritis and reviews cases involving arthritis, discitis, and osteomyelitis in the context of *S. moniliformis* infections (21–65). It aims to demonstrate the specific circumstances and clinical features that should raise suspicion of *S. moniliformis* osteoarticular infection (OAI), further delve into the pathophysiology, and explore how microbiological diagnosis and treatment could be optimized.

## Case description

In August 2023, an 83-year-old Belgian man presented at Erasme tertiary hospital in Brussels with fever and functional impairment of his right knee. The medical history revealed that 3 days prior to admission, the patient's left index finger was inflamed and painful. The following day, he noticed spontaneous improvement in his left hand but experienced pain in his right knee, even though he had no history of either knee trauma or pain. On the 3^rd^ day, he developed a fever of 39°C with chills, his knee had become swollen and the pain worsened to the point where he could not walk, prompting him to go to the hospital.

Physical examination showed a swollen and very painful knee. In contrast, the finger joint displayed redness and warmth but with only mild pain and swelling. The patient had a recorded fever of 38.2°C. When questioned, he denied any recent travel abroad, walking in the forest, or insect bites. The patient had several underlying medical conditions, including hormone replacement therapy for Hashimoto's thyroiditis, Biermer's anemia, and was treated for hypertension. In 2012, he underwent prosthetic aortic replacement.

Left hand and right knee X-rays revealed moderate rhizarthrosis in the hand and severe knee osteoarthritis with intra-articular effusion. Initial blood tests showed moderate acute kidney failure, with a creatinine clearance calculated with the CKD-EPI equation (66) of 43 mL/min/1.73 m^2^ (normal range: ≥90 mL/min/1.73 m^2^) and a biological inflammatory response, including an increased white blood cell count (WBC; 10, 860 cells/mm^3^) with 79% neutrophils and elevated C-reactive protein (CRP; 200 mg/L). Before the initiation of antibiotics, two sets of blood cultures were collected and incubated using the BD BACTEC FX™ blood culture system (Becton, Dickinson and Company, USA). Additionally, fluid was aspirated from the right knee joint and inoculated in a BD BACTEC™ Peds Plus™ bottle. The cell count of this fluid revealed a high WBC count (178, 816/mm^3^) with a predominance of neutrophils (89%) and 4, 200 red blood cells (RBC)/mm^3^. The fluid total protein level was elevated (38 g/L). No crystals were detected and the uric acid fluid level was within the normal range (59 mg/L). Empirical antibiotic therapy with ceftriaxone (2 g twice daily) and flucloxacillin (2 g every 4 h), was promptly initiated. An arthroscopic washout procedure was performed and a second sample of joint fluid was collected that was consistent with the first one (88, 704 WBC/mm^3^, 91% neutrophils, 28, 200 RBC/mm^3^, no germ or crystal were observed by direct examination). The culture was initiated using sheep blood agar (BD™ Columbia Agar with 5% Sheep Blood, BD), chocolate agar (Chocolate PolyViteX™ agar, Biomerieux), and Schaedler broth (BBL™ Schaedler Broth with Vitamin K1, BD) incubated at 35 °C with 5% CO_2_, and a BD BACTEC™ Peds Plus™ bottle.

**Figure 1:**
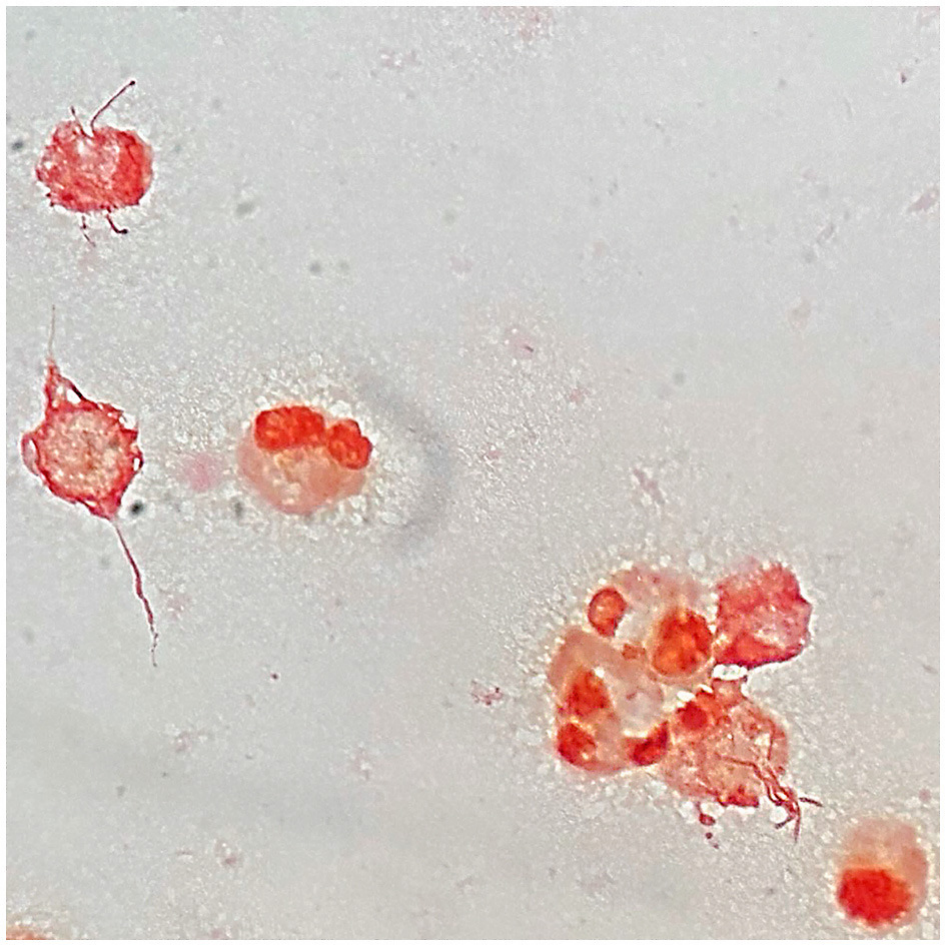
Microscopic image of a Gram-stained smear of *Streptobacillus moniliformis* from knee joint fluid culture in BD BACTEC™ Peds Plus™ bottle when growth was detected by BD BACTEC FX blood culture system, demonstrating pleomorphic and irregular Gram-negative rods in contact with neutrophils and also debris. Magnification, × 1000.

After 29 h of incubation, growth was detected in the BD BACTEC™ Peds Plus™ bottle containing the joint fluid that was initially sampled at the emergency department. The Gram-stained smear revealed pleomorphic and irregular Gram-negative rods (Figure 1). Subsequent subcultures on blood and chocolate agars plus Schaedler broth were performed.

**Figure 2:**
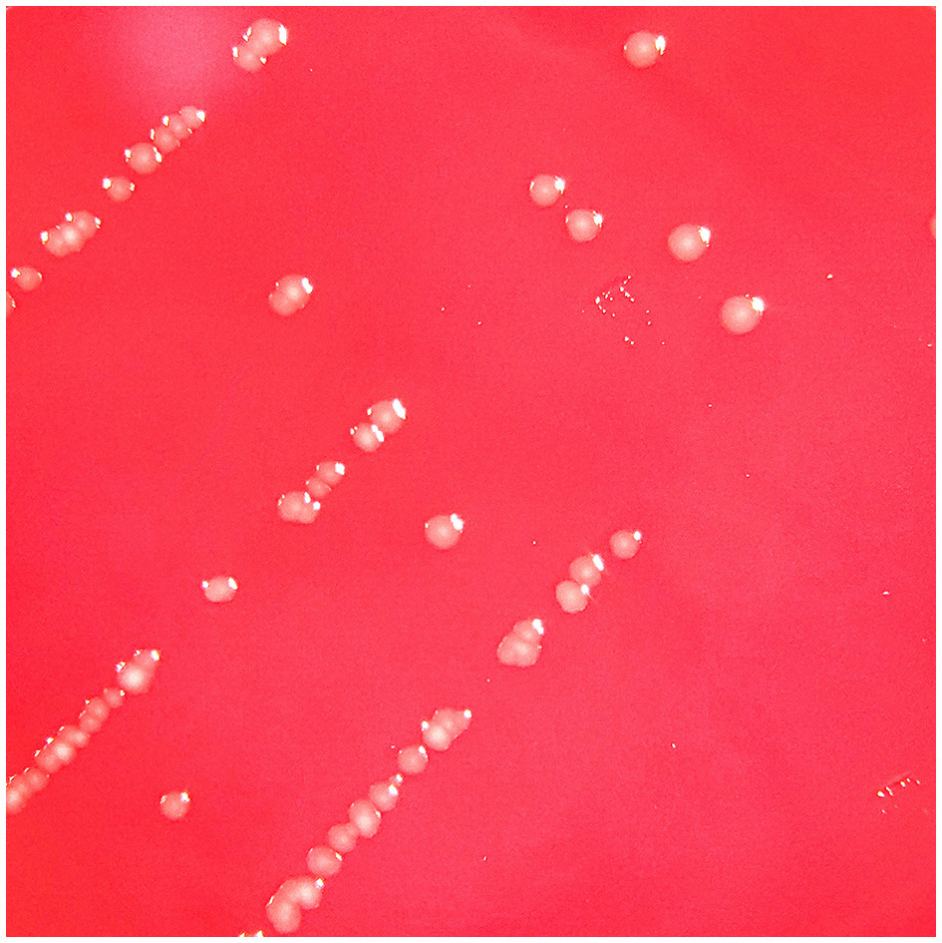
Three-day culture in capnophilic atmosphere on 5% sheep blood agar (BD™ Columbia Agar with 5% Sheep Blood, BD) showing tiny, non-hemolytic, pale gray, smooth and slightly shiny colonies.

**Figure 3:**
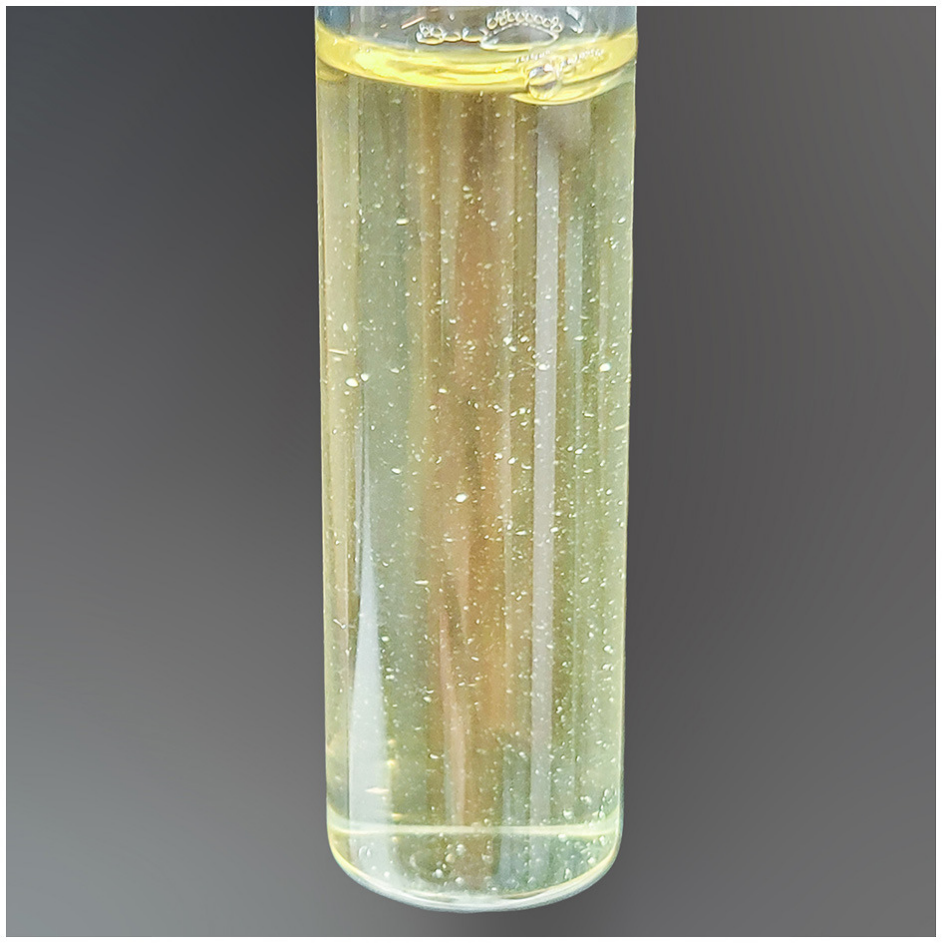
Three-day culture in capnophilic atmosphere in BBL™ Schaedler Broth with Vitamin K1, BD, showing the typical “puffball” growth characteristic of *Streptobacillus moniliformis* after gentle agitation of the broth.

On the third day, the patient reported feeling more comfortable. His knee was less inflamed and less painful, while his hands showed nothing remarkable. An infectious disease specialist was consulted leading to the discontinuation of flucloxacillin. Upon further inquiry by the specialist, the patient recalled being bitten on the right hand by a rat brought home by his cat approximately 10 days before the onset of his symptoms. In the meantime, weak growth was observed on the blood agar plate corresponding to the subculture from the bottle that had been positive the day before. This growth evolved, developing small, pale gray, shiny, round-shaped non-hemolytic colonies after 48 h of incubation (Figure 2). The microorganism was identified as *S. moniliformis* with a score > 2.3 using matrix-assisted laser desorption ionization-time of flight mass spectrometry (MALDI-TOF MS; Biotyper Sirius IVD version 4.2.100; Bruker Daltonics, Germany). Furthermore, a 1421 bp PCR sequencing (GenBank accession no. PP350726.1) of the 16S rRNA gene on the isolate confirmed the identification of the species. The genetic sequence exhibited a 100% match with *S. moniliformis* DSM12112^T^ (GenBank accession no. CP001779) but only a 98.59% match with *S. notomytis* AHL_370–1^T^ (GenBank accession no. KR001919) and < 98% identity to other *Streptobacillus* species in the EzBioCloud 16S database (http://www.ezbiocloud.net/eztaxon). Cultures on chocolate agar plates, blood cultures and the culture of the second knee fluid remained sterile. Similarly, attempts to conduct antimicrobial susceptibility testing by strain subculture on Mueller Hinton agar with 5% horse blood and 20 mg/L ß-NAD did not yield any results. However, the strain subculture in Schaedler broth displayed the characteristic “puffball” growth pattern associated with *S. moniliformis* (Figure 3).

**Figure 4:**
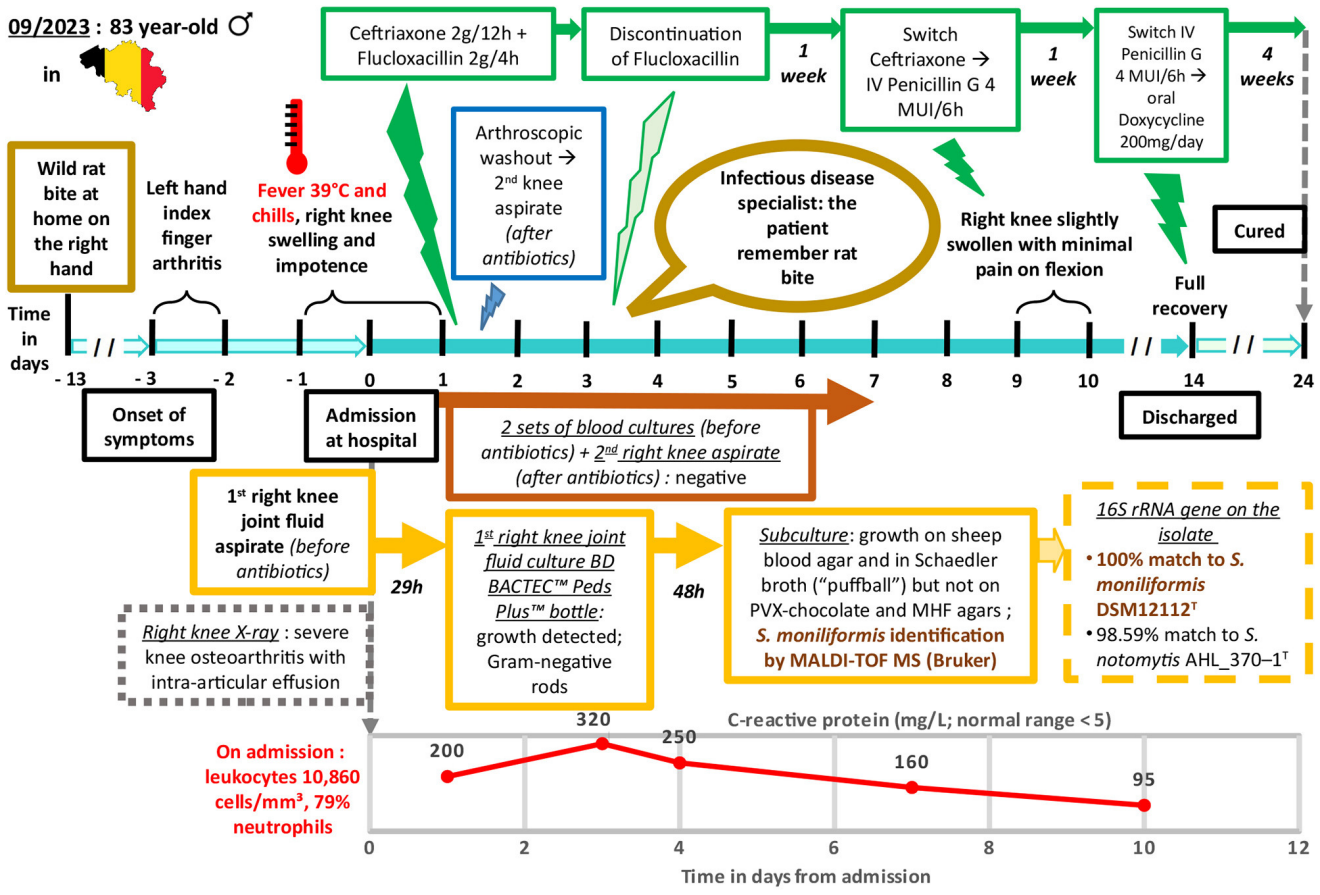
Relevant data from the episode of care organized as a timeline.

With the patient showing significant clinical improvement by the 9^th^ day, ceftriaxone was replaced with penicillin G at a dosage of 4 million international units every 6 h. After 2 weeks of intravenous antibiotic therapy, the patient had fully regained the range of motion in his knee which no longer caused him pain. Consequently, he was discharged from the hospital with oral doxycycline (200 mg/day) for four supplementary weeks. The patient was observed at the end of antibiotic treatment to have a sustained complete recovery. Figure 4 shows a timeline of relevant data from the episode of care.

## Discussion

RBF has been characterized as an emerging disease for more than two decades (15, 67). However, in our laboratory, which annually conducts over 800, 000 bacteriological analyses for several hospitals in Brussels, Belgium, we have only identified *S. moniliformis* twice over the past decade, including the present case (64). Importantly, no significant outbreaks have been reported in the literature since the end of the 20^th^ century. A search on PubMed using keywords such as “RBF,” “*Streptobacillus*,” “arthritis,” “discitis,” and “osteomyelitis” yielded only 44 sporadic case reports since 1985, along with one case potentially linked to a small intrafamilial outbreak (39) (Supplementary Table S1). Despite the increasing awareness of this disease and the rising number of people keeping rats as either pets (68) or breeding rats to feed pet snakes (69), *S. moniliformis* infections seem to remain exceptionally rare.

However, the prevalence of *S. moniliformis* OAI and more broadly of RBF and its complications is likely underestimated. The first challenge in diagnosing RBF is the potential for delayed onset of symptoms. While the estimated incubation periods for both HF and RBF are < 1 week (7, 10, 15), it can extend to 3 weeks in RBF cases (16, 17, 21). Among the cases reviewed, 21 individuals had been bitten by rats, and of those, seven might have been bitten more than 1-to-3 weeks before the onset of their symptoms, which accounts for 36% (*n* = ^8^/_22_) of cases including our patient (21, 28, 34, 36, 54, 56, 59) (Supplementary Table S1). Second, in 36% (*n* = ^16^/_45_) of cases, rodent exposure is only mentioned by the patient after *S. moniliformis* detection (Supplementary Table S1). Recognizing this rare condition is therefore difficult in the presence of non-specific symptoms such as fever, skin rashes, and polyarthralgias, mimicking an ordinary viral infection (24, 59). Moreover, as at least HF resolves spontaneously in many cases (7, 39), patients may generally not seek medical care, leading to an overrepresentation of severe cases in the literature.

As its name suggests, fever, which was also observed in our patient, appears to be the symptom most commonly associated with local signs of OAI among the 45 cases reviewed in this case report. Only eight patients (18%) did not exhibit either fever or hypothermia prior to or on admission (22, 32, 35, 36, 39, 41, 57, 62) (Supplementary Table S1). Among them, only two had underlying conditions that could compromise their immunity, namely diabetes mellitus (39) and rheumatoid arthritis (RA) which was treated with methotrexate and tocilizumab (57). Conversely, six patients with diabetes mellitus (50) and RA treated with steroids and methotrexate (48), and alcoholism (*n* = 3) (27, 42, 65) or HIV with AIDS (44) still presented fever. In total, 82% of patients (*n* = ^37^/_45_) were without immunodeficiency (Supplementary Table S1). For 56% of patients (*n* = ^25^/_45_) no underlying conditions were reported.

The precise pathophysiology of RBF has currently not been fully elucidated (21, 37, 40, 61, 64, 65). Although “acute septicemic erythema multiforme” and “erythema arthriticum epidemicum” were initially used to describe RBF and HF (6, 7), respectively, skin rashes were observed only in 42% of the cases included in this study (*n* = ^19^/_45_) (Supplementary Table S1). The mean age of the patients with rash was 29 years whereas the mean age of patients without skin symptoms was 56 years. Notably, the patient of the present case did not display any cutaneous manifestations. Despite the historical characterization of HF and RBF based on a typical triad of symptoms (7, 10, 17, 19, 21, 40, 70), only 40% (*n* = ^18^/_45_) exhibited all three manifestations (Supplementary Table S1) consistently with fewer reviews published in the last 30 years that have examined RBF among adults (17) and children (16, 18) and *S. moniliformis* OAI (21, 22, 40, 51) and endocarditis (28, 71).

The mechanisms responsible for arthritis in the context of RBF also remain controversial (21, 37, 40, 61, 64). While many authors have supported the diagnosis of septic arthritis (25, 26, 30, 33, 41, 42, 44, 45, 47, 56, 58–60, 62–64), others have speculated that the joint involvement in RBF could be at least partially attributed to either autoimmune or reactive arthritis (21, 40, 64). Despite autoimmune antibody testing reported in 16 out of 45 cases, positive results were obtained in only two cases (61, 65). The authors concluded that *S. moniliformis* triggered the production of these antibodies, as observed in other infections. Unfortunately, in the first case, arthrocentesis was performed after ceftriaxone initiation, and, although the joint fluid was purulent, the culture remained sterile. In the second case, no joint sample was collected. In addition, among the 45 reviewed cases, only three patients had underlying autoimmune diseases (RA; *n* = 2; psoriasis) (48, 55, 57) (Supplementary Table S1), suggesting that it is not a determining factor for developing *S. moniliformis* OAI. This aligns with the patient in the present case report, who had either no family or personal history of rheumatological disease.

Joint fluid analyses were conducted in 37 out of the 45 cases. Detailed cytology data were available for 17 patients. The WBC count per mm^3^ ranged from 14, 000 to 104, 000, with at least 80% of neutrophils except for one sample (63). These values, similar to those of our patient, are consistent with the cutoff values presented in the literature for OAI (72, 73). In 10 more cases joint fluids were described as purulent (*n* = ^9^/_45_) and/or contained numerous neutrophils (*n* = ^5^/_45_) (Supplementary Table S1).

Gram stain examinations were reported in 30 cases (Supplementary Table S1). Gram-negative rods were observed in nine cases whereas other morphologies were observed in five fluids, highlighting the limited sensitivity and specificity of this examination. It is worth noting that the bacterium's morphology, characterized by numerous lateral bulbous swellings, can resemble a string of pearls, which explains its species designation “*moniliformis*” derived from the Latin word for “*necklace*” (74). However, due to its irregular appearance, it can easily be mistaken for protein debris.

In 71% of cases (*n* = ^24^/_34_) where synovial fluid culture was performed, *S. moniliformis* was isolated on either 5% sheep or horse blood agar media and in either cooked meat or thioglycolate broth at 35°C typically after 48 to 72 h of incubation (Supplementary Table S1). In four of these cases, the primary isolation was reported to have been obtained under aerobic conditions enriched with 5 to 8% CO_2_ (33, 40, 45, 64), whereas in four cases it was reported to have been obtained under anaerobic conditions (26, 33, 58, 59) and in one case under microaerophilic conditions (27). Otherwise, according to Eisenberg et al. (74) initial cultivation from clinical samples was optimal in the presence of 5–10% CO_2_ but grow only weakly anaerobically. Of the 10 negative cultures (28, 39, 41, 44, 47, 50–53, 57), four were performed after the start of antibiotic therapy (39, 41, 50, 57) and one was performed on MacConkey agar (52), on which *S. moniliformis* cannot grow. In the present case report, *S. moniliformis* was sub-cultured from a positive pediatric bottle inoculated with synovial fluid under both anaerobic and aerobic atmospheres either with or without 5% CO_2_ enrichment on Columbia media enriched with hemin or 5% sheep blood plus yeast extract and peptic digest of animal tissue, but not on Chocolate PolyViteX media containing 5% sheep blood plus meat peptone only (Supplementary Table S2). Excluding these five improperly performed cultures, *S. moniliformis* was identified in 25 cases by culturing and twice by direct 16S rRNA PCR sequencing on fluids (44, 47), giving a detection rate of 90% (*n* = ^27^/_30_). Interestingly, *S. moniliformis* was also detected in all four fluids in which the 16S rRNA gene was directly sequenced (41, 44, 47, 57). These results provide strong evidence for the pyogenic nature of the osteoarticular symptoms in RBF rather than suggesting either autoimmune or reactive arthritis. Furthermore, considering that *S. moniliformis* can grow on commonly used commercial 5% sheep or horse blood-enriched agar and plain broth in a capnophilic atmosphere, it is our belief that the main obstacle to detecting it in joint fluid is the failure to collect fluid or the administration of antibiotics prior to sampling. In cases where there is clinical suspicion of OAI with suggestive cytology but negative culture, direct 16S rRNA gene sequencing to detect the genus *Streptobacillus* and its related species should also be considered. In addition, there were 17 cases of growth of *S. moniliformis* in blood culture bottles. In some cases it was stated that growth was observed in either adult (39, 48, 51, 54, 60), pediatric (63) bottles, under aerobic (60, 63), or anaerobic (39, 48, 51, 54) atmosphere. In three of these cases it was mentioned that subculturing *S. moniliformis* from blood bottles was achieved using capnophilic atmosphere enriched with either 5 or 10% CO_2_ (48, 52, 54). However, in one of the latter, subcultures were also obtained in anaerobic and unenriched aerobic conditions (48). These results are again consistent with Eisenberg et al.'s (75) revised description of the genus *Streptobacillus* (6), which states that most strains require a capnophilic atmosphere containing 5%−10% CO_2_ and grow weakly anaerobically, but few strains can grow aerobically (74).

Furthermore, it is remarkable that MALDI-TOF MS provided excellent strain identification and is even considered as the new gold standard for species discrimination by Eisenberg et al. (74). However, spectra of the recently described related species *S. notomytis* (75), *S. ratti* (76), *S. felis* (77), *S. canis* (78), and *Pseudostreptobacillus hongkonensis* (79, 80) are currently not included in the IVD Bruker 2024 database, which could lead to misidentification. Nevertheless, in 2016 Eisenberg et al. (74) claimed that spectra of all members of the genus *Streptobacillus* are available through MALDI-UP, a free dedicated database platform for users (81). An alternative method for confirming species identification remains PCR gene sequencing. However, Eisenberg et al. (74) again specified that to unambiguously identify species within the genus *Streptobacillus*, especially in the closely related species *S. moniliformis, S. felis, S. notomytis*, and *S. ratti*, identification based on the 16S rRNA gene sequence should always be confirmed by sequencing other gene loci such as *groEL, recA*, and *gyrB*. Sequencing results should also be considered with references to the fact that *R. norvegicus* seems to be the only carrier of *S. moniliformis* (82, 83) and seems not to host other *Streptobacillus* species (84–86).

To further explore the pathophysiology of *S. moniliformis* infections, we focused on its response to anti-inflammatory drugs (AID) and antibiotics. In the present case report, the 14 patients who received AID before diagnosis experienced clinical deterioration, particularly related to arthritis (21, 25, 27, 28, 34, 36, 37, 39, 42, 48, 51, 53, 62, 65), while after receiving antibiotics all patients showed quick improvement and recovery (*n* = 44) (Supplementary Table S1). The drug response once again supports that the osteoarticular symptoms of RBF are not the result of an excessive immune response.

*Streptobacillus moniliformis* is known to be highly susceptible to β-lactams, vancomycin, tetracycline, and clindamycin (27, 28, 83, 87). Typically, patients are treated with either β-lactams, mostly penicillin G, ceftriaxone or amoxicillin (-clavulanate) (Supplementary Table S1). When oral switching is performed, the regimen consist mainly of either amoxicillin (-clavulanate) (31, 35, 45–47, 54, 57, 60, 61, 63) or doxycycline (34, 44, 64) (Supplementary Table S1).

Despite the occasional delay in diagnosis, the prognosis for RBF is excellent. Seventy-two percent of patients, including the patient in the present case report, achieved full recovery at the end of follow-up (Supplementary Table S1). Other patients experienced significant symptom improvement, with minimal residual pain and limited range of motion. There was one exceptional case of a infant who, due to an initial assumption of a viral infection, did not receive antibiotics and tragically passed away 3.5 days after symptom onset (24), resulting in a mortality rate of 2.2%, (*n* = ^1^/_46_) (Supplementary Table S1), which is notably lower than the mortality rates reported in previous studies (15, 17, 70, 83, 88). Nearly all fatal cases were attributed to either endocarditis (13, 89–98), pericarditis (99) or RBF among infants (24, 100).

## Conclusion

Although rare, when faced with symptoms of recurrent fever and septic arthritis and/or discitis, *S. moniliformis* infection should be considered, even when the patient does not report direct rat exposure, especially if arthritis is migratory and involves multiple joints. Rash was present in over 80% of cases involving children but was observed in only 20% of patients aged 30 or more years. Joint fluid culture in capnophilic atmosphere prior to antibiotic therapy on broadly used 5% sheep blood Columbia agar and thioglycolate or Schaedler broth should lead to the isolation of *S. moniliformis*. MALDI-TOF MS can be effective in identifying strains, but comparison of spectra with those of all members of the genus *Streptobacillus* available on either the MALDI-UP platform or multilocus gene sequencing is required to confirm species identification. If the culture remains negative, direct 16S rRNA PCR may be helpful. Treatment should include β-lactams. Excluding infants and endocarditis, the prognosis for *S. moniliformis* infection appears good, although some cases probably remain undiagnosed, leaving an incomplete picture of outcomes.

## Data availability statement

The original contributions presented in the study are included in the article/Supplementary material, further inquiries can be directed to the corresponding author.

## Ethics statement

Ethical approval was not required for the study involving humans in accordance with the local legislation and institutional requirements. Written informed consent to participate in this study was not required from the participants or the participants' legal guardians/next of kin in accordance with the national legislation and the institutional requirements. Written informed consent was obtained from the individual(s) for the publication of any potentially identifiable images or data included in this article.

## Author contributions

EG: Writing – original draft, Writing – review & editing. EL: Investigation, Writing – review & editing. SW: Investigation, Writing – review & editing. MD: Investigation, Writing – review & editing. NY: Investigation, Writing – review & editing. MH: Writing – review & editing. DM: Supervision, Writing – review & editing.
